# Clinical Profiling and Biomarkers for Post-Operative Atrial Fibrillation Prediction in Patients Undergoing Cardiac Surgery

**DOI:** 10.3390/jcm12103565

**Published:** 2023-05-19

**Authors:** Diego Iglesias-Álvarez, Xiaoran Fu, José Manuel Martínez-Cereijo, Rosa María Agra-Bermejo, Sonia Veiras-Del Río, Salomé Selas-Cobos, María Victoria Rial-Munin, María Eiras-Mariño, Adrián Martínez-Salgado, Manuel Taboada-Muñiz, Laura Reija-López, Souhayla Souaf, Javier García-Carro, Ángel Luis Fernández-González, Belén Adrio-Nazar, José Ramón González-Juanatey, Sonia Eiras, Moisés Rodríguez-Mañero

**Affiliations:** 1 Department of Cardiology, Complexo Hospitalario Universitario de Santiago, 15706 Santiago de Compostela, Spain; 2 Traslational Cardiology, Health Research Institute of Santiago de Compostela (IDIS), 15706 Santiago de Compostela, Spain; 3 CIBERCV—Centro de Investigación Biomédica en Red de Enfermedades Cardiovasculares, 28029 Madrid, Spain; 4 Department of Cardiac Surgery, Complexo Hospitalario Universitario de Santiago, 15706 Santiago de Compostela, Spain; 5 Department of Anaesthesia and Critical Care, Complexo Hospitalario Universitario de Santiago, 15706 Santiago de Compostela, Spain

**Keywords:** post-operative atrial fibrillation, cardiac surgery, inflammation, adiposity, biomarkers, orosomucoid

## Abstract

Post-operative atrial fibrillation (POAF) is the most common arrhythmia in the post-operative period after cardiac surgery. We aim to investigate the main clinical, local, and/or peripheral biochemical and molecular predictors for POAF in patients undergoing coronary and/or valve surgery. Between August 2020 and September 2022, consecutive patients undergoing cardiac surgery without previous history of AF were studied. Clinical variables, plasma, and biological tissues (epicardial and subcutaneous fat) were obtained before surgery. Pre-operative markers associated with inflammation, adiposity, atrial stretch, and fibrosis were analyzed on peripheral and local samples with multiplex assay and real-time PCR. Univariate and multivariate logistic regression analyses were performed in order to identify the main predictors for POAF. Patients were followed-up until hospital discharge. Out of 123 consecutive patients without prior AF, 43 (34.9%) developed POAF during hospitalization. The main predictors were cardiopulmonary bypass time (odds ratio (OR) 1.008 (95% confidence interval (CI), 1.002–1.013), *p* = 0.005), and plasma pre-operative orosomucoid levels (OR 1.008 (1.206–5.761). After studying differences regarding sex, orosomucoid was the best predictor for POAF in women (OR 2.639 (95% CI, 1.455–4.788), *p* = 0.027) but not in men. The results support the pre-operative inflammation pathway as a factor involved in the risk of POAF, mainly in women.

## 1. Introduction

In the post-operative cardiac surgical setting, new-onset atrial fibrillation (AF) is not a benign entity, as it is known to increase the risk of all-cause mortality, stroke and critical care unit, and overall hospital length of stay. This complication is estimated to occur in nearly one-third of the patients undergoing open heart surgery [1,2]. Some pre-operative and peri-operative strategies were recommended in order to decrease the risk of post-operative atrial fibrillation (POAF) with mixed results [3]. This is the case of pretreatment with drugs (beta blockers, amiodarone), electrolyte infusions, and different anesthetic or surgical techniques (systemic temperature control, coated circuits, posterior pericardiotomy, and off-pump bypass grafting) among others. Meanwhile, other studies focused their attention on the cellular and molecular level, such as modulation of the neutrophils and inflammatory pathways or modulation of the nervous system through botulin toxin on epicardial fat [4,5]. Many triggers and substrates can promote acute AF during procedures and personalized management may be required for reducing its incidence and recurrence [6].

Relevantly, predisposing conditions and long-term management of this arrhythmia may differ between the medical and the surgical patients, as the evidence in the latter is significantly scarcer. Particularities of POAF following cardiac surgery and its differences with other types of AF are not completely clarified yet, but the direct surgical trauma to the heart, inflammation following cardiopulmonary bypass, and increased sympathetic stimuli may be involved [7]. As such, a better understanding of the pathways involved in POAF might help to improve its management.

On top of that, there is a growing interest in the study of internal signaling pathways that can alter tissue homeostasis. Inflammatory and pro-thrombotic response mechanisms, metabolic or hormonal disarrangements, and fibrosis promote ventricular and atrial remodeling. Thus, some circulating markers of atrial strain and function [8], adiposity [9], cytokine-induced stress [10], and inflammation [11] were found to be associated with AF.

The aim of our study was to investigate the association of local and peripheral markers involved in inflammation, strain, adiposity, and fibrosis with the development of POAF in the post-operative period. We studied clinical and echocardiographic parameters, and biochemical and molecular markers on local tissues (subcutaneous and epicardial fat), peripheral cells, and blood.

## 2. Materials and Methods

### 2.1. Study Design and Population

This is a prospective, observational, and single-center cohort study conducted at the University Hospital of Santiago de Compostela (a university-affiliated tertiary center in Santiago de Compostela, Spain). Between August 2020 and September 2022, consecutive patients undergoing cardiac surgery (coronary, valve, or mixed surgery) under informed consent were eligible for inclusion. The exclusion criteria were prior open-heart surgery, prior history of AF or severe active infective disease, or cardiogenic shock at hospitalization.

The protocol was approved by Galician Clinical Ethics Committee and followed the Declaration of Helsinki’s rules. As per protocol, all patients undergo routine evaluation before cardiac surgery. This includes a minimum of medical history assessment, clinical examination, electrocardiography, laboratory blood sampling, and echocardiography performed by cardiologists specifically dedicated to cardiac imaging and anesthetic evaluation.

At the operating room, after sternotomy, epicardial and subcutaneous fat biopsies (0.2–0.5 g) were obtained and stored at −80 °C for RNA expression levels. Blood samples into tubes with heparin or EDTA were obtained before starting the surgical procedure.

After the operation, the patients were transferred to the intensive care unit (ICU) for recovery. From there, when deemed suitable, discharged to the standard cardiac surgical ward or cardiology intermediate care unit. Patients were monitored via continuous ECG telemetry while in the intensive or intermediate care unit. Daily 12-lead ECGs were recorded routinely until the day of discharge. Further ECGs could be requested physician in charge if considered necessary as per usual clinical practice. The follow up period for this study finished after hospital discharge.

### 2.2. Study Primary End-Point

The primary end-point was the development of AF as defined by the European Society of Cardiology guidelines [12] (a cardiac rhythm with the absence of distinct *p* waves and irregularly irregular RR intervals). This could be documented either in the continuous ECG monitoring, in a 12-lead ECG, or both, depending on whether the patient was under cardiac telemetry or not during the event.

### 2.3. Clinical, Echocardiography, and Biochemical Variables (Pre- and Peri-Operative)

#### 2.3.1. Clinical Variables

Demographic and anthropometric variables, previous clinical diagnoses, and chronic medications were recorded after medical history taking and reviewing the electronic history records.

#### 2.3.2. Echocardiography

Transthoracic echocardiography was routinely performed during hospitalization (or in the outpatients’ clinic for scheduled procedures) by trained clinicians in a comprehensive way, initially during the pre-operative evaluation and again after heart surgery. Measured parameters included left ventricular dimensions, ejection fraction (as calculated by the international Simpson’s method), left and right atrial dimensions, right ventricular dimensions, contractility (TAPSE, S’), pulmonary pressures, and valvular heart diseases (significant stenosis or regurgitation).

#### 2.3.3. Pre-Operative Clinical Laboratory Variables

Blood cell count, renal and liver function tests, electrolytes, coagulation, and metabolic profile (HbA1C, total cholesterol, and LDL-c), as well as NTproBNP levels, were determined as per routine practice.

#### 2.3.4. Pre-Operative Circulating Blood Biomarkers

Fasting blood samples were collected in EDTA tubes in the operating room before the surgical incision. After being centrifuged at 1800× *g* for 10 min, plasma was stored at −80 °C. Selected circulating markers of atrial strain (atrial natriuretic peptide), adiposity (fatty acid binding protein (FABP4) and leptin), cytokine-induced stress (growth differentiation factor 15 (GDF15)), complement (C5a), and matrix extracellular (Thrombospondin-2) were analyzed by magnetic Luminex multiplex test kit (R&D Systems, Minneapolis, MN, USA). The manufacturer’s instructions were followed and the plasma was diluted twice.

Alpha1-acid glycoprotein, also known as orosomucoid (ORM), was measured as a marker of inflammatory activity [13]. ORM levels were determined by enzyme-linked immunoassay (ELISA) with a detection limit of 59 ng/mL (SEA816Hu, Cloud Clone). Diluted plasma 1:1000 was used for this determination.

#### 2.3.5. Pre-Operative Neutrophils Migratory Activity

Fasting blood samples were also collected into lithium heparin-coated vacutainers transferred and processed in the laboratory within the first hours. Neutrophils were isolated by single-step centrifugation of whole blood onto Polymorphprep (Proteogenix, Schiltigheim, France) following the manufacturer’s recommendation. Blood was pipetted on Polymorphprep (1:3) and centrifuged at 500× *g* for 35 min without brake. Granulocytes, mainly neutrophils were carefully taken and resuspended in RPMI 1640 media supplemented with 25 mM HEPES (Lonza Biologics, Porriño, Spain). After washing, the neutrophils number was determined by Scepter™ 2.0 Cell Counter (Millipore^®^, Merck Life Science S.L.U., Madrid, Spain). Four hundred thousand neutrophils between 9–12 µm were used for migration assays. They were seeded into transwells with 3 µm in size (Merck Life Science S.L.U., Madrid, Spain). Migrated cells were determined after treatment with or without complement component 5a (C5a) at 11 nM for 90 min. Migrated cells were detached with EDTA (0.05M) for 15 min at 4 °C. Afterward, migrated and non-migrated neutrophils were collected and centrifuged at 500× *g* for 5 min and lysed for DNA quantifying using CyQUANT^®^ GR dye (Thermo Fisher Scientific, Waltham, MA, USA), according to the manufacturer’s protocol. After that, fluorescence that represents also migrated and no migrated neutrophils, was recorded at an excitation/emission wavelength (485/525 nm) with (FLUOstar OPTIMA, BMG Labtech, Allmendgrün, Germany). Results were expressed as a percentage of migrated total cells in relative fluorescence units (RFU) as it was described before [14].

#### 2.3.6. Pre-Operative Neutrophils, Monocytes, Epicardial Fat, and Subcutaneous Fat mRNA Expression Analysis

Isolated neutrophils and monocytes from blood, epicardial fat, and subcutaneous fat biopsies were lysed and RNA was isolated by AllPrep DNA/RNA/Protein Mini Kit (Qiagen, Hilden, Germany). After retro-transcription, using the Maxima First Strand cDNA Synthesis Kit (Thermo Fisher Scientific, Waltham, MA, USA), 1 μL of cDNA was used for amplifying the enzymes (MPO, DEFA3, MPO, NGAL, LF), and adhesion molecules (CXCR2, CD11B, SELL, CXCR4) from neutrophils and monocytes or adiposity (FABP4, CD36), inflammatory cells (CD14, CD16, CD3, CD68), fibroblasts (COL1A2, PREF-1), and myofibroblasts (a-SMA) markers in epicardial and subcutaneous fat as it was previously described [15].

#### 2.3.7. Peri-Operative Clinical Laboratory Variables

Cardiopulmonary bypass (CPB) time during surgery was measured in all patients. After surgery, daily blood tests with blood cell count, renal and liver function tests, electrolytes, and coagulation profiles were obtained. Additionally, and according to the patient’s clinical situation, arterial and mixed venous blood gases could be ordered to measure pH, lactate, and SvO_2_.

#### 2.3.8. Peri-Operative Clinical Variables

Time on mechanical ventilation, the requirement of vasopressors or inotropes and days of support, length of stay in the ICU, and complications during the hospitalization were recorded.

### 2.4. Statistical Analysis

Continuous variables were reported as numbers and categorical variables as percentages. To check the normal distribution of variables, Kolmogorov–Smirnov tests were performed. Normal variables are presented as mean ± standard deviation (SD). Scattered variables are represented as interquartile ranges. Differences in continuous variables between patients with and without POAF were determined by the Student’s *t*-test or Mann–Whitney according to the normality of data. Categorical variables were compared using χ^2^ test.

Univariate and multivariate logistic regression analyses were performed. We selected EUROSCORE II, CPB duration, and preoperative ORM values as independent variables: EuroSCORE II is a surgical risk model created to predict surgical mortality and is commonly used worldwide as a pre-operative evaluation tool. It includes important clinical, echocardiography, and laboratory parameters and surgical variables. CPB duration and preoperative ORM values are associated with the inflammatory hypothesis, which is being investigated as a contributor to POAF. The left atrial area was not included as differences between both groups were not clinically significant. Finally, some of the biomarkers were not considered appropriate for the multivariate model as their values were not available for all the patients.

Hosmer–Lemeshow test was used for testing the goodness of fit for regression. Cut-off values were determined by the area under the curve (AUC).

All statistical analyses were performed by SPSS Statistics 26 (IBM, Armonk, NY, USA).

## 3. Results

Overall, 123 consecutive patients with no prior history of AF undergoing heart surgery were included. The general baseline characteristics of the population are described in Table 1. Of note, a significant number of patients were pre-treated with anti-platelet agents, beta-blockers, ACE-I/ARB, and statins. During the post-operative period, 43 patients (34.9%) developed POAF. Details about complications during the peri-operative course are displayed in Table 2.

Regarding clinical variables between those who developed and those who did not develop POAF, there were statistically significant differences in predicted surgical risk (as in EuroSCORE II), left atrial dimensions, and CPB time during surgery.

Differences in biomarkers were as follows: the local epicardial fat analysis showed higher expression of fatty acid binding protein 4 (FABP4) mRNA levels in those who developed POAF (*p* < 0.05). These differences were not found in subcutaneous fat biopsies.

Peripheral neutrophils also showed higher expression levels of CD16, a marker of mature neutrophils (*p* < 0.01) in those patients who developed POAF. There were no changes in its migratory activity. Monocyte transcriptome did not differ between these two groups of patients.

Plasma circulating markers of adiposity, stretch, or fibrosis were not different between patients with and without POAF. However, ORM was higher in those who developed POAF (*p* < 0.05). Although insulin levels did not reach the statistical difference between patients (*p* = 0.098), they were also higher in those who suffered POAF. Table 3 shows the differences in terms of clinical variables and biomarkers between patients who developed POAF or not, whereas Table 4 shows the same information specified by sex.

Our results showed in the univariate and multivariate models that the main risk variables for POAF were CPB and preoperative ORM levels (Table 5).

**Figure 1:**
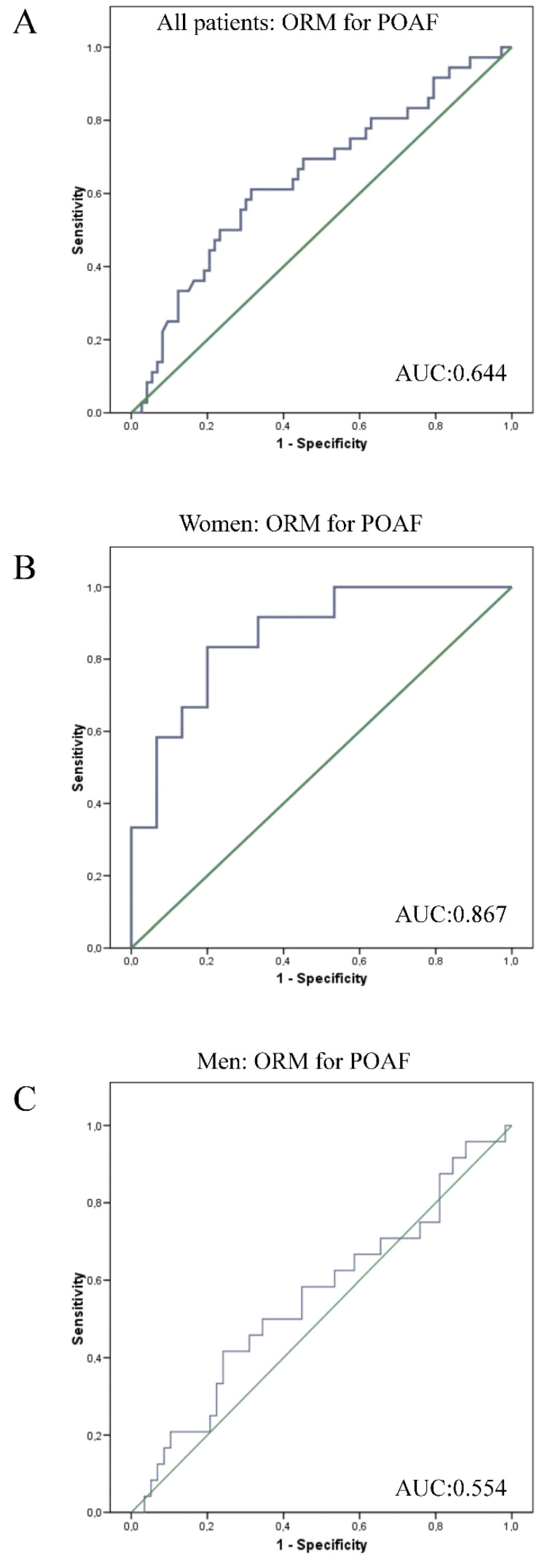
(**A**). ROC curves, ORM for POAF in all patients. (**B**). ROC curves, ORM for POAF in women. (**C**). ROC curves. ORM for POAF in men.

We also performed an area under the curve (AUC) for analyzing the degree of separability between the two groups (with and without POAF) in terms of some of the previously mentioned markers. We found the best AUC for ORM (0.644, 95% CI 0.542–0.756, *p* = 0.009) (Figure 1A).

### Sexual Dimorphism and Markers

Although the population was limited and the statistical power might be affected, we tried to define the markers for POAF in women and men. Out of 30 female patients, 14 (46.6%) developed POAF as compared to 29 out of 93 (31%) in men. None of the clinical characteristics differed between both groups. Regarding biomarkers, significant differences were observed for circulating (FABP4 and leptin) and epicardial fat adiposity markers (FABP4 and CD36). In women, the main clinical differences between those developing POAF were CPB time and previous admission due to heart failure. ORM plasma levels were also significantly higher whereas the local inflammatory cells on epicardial fat were reduced. AUC for ORM was 0.844 (95% CI 0.668–1.021; *p* = 0.01) (Figure 1B). Multivariate logistic regression analyses on the female population showed that high ORM was the strongest independent variable for POAF risk (OR 2.639, 95% CI 1.455–4.788, *p* < 0.05) (Table 5). This was not the case in men where ORM was not associated with POAF occurrence.

## 4. Discussion

In our cohort of post-operative cardiac surgical patients without previous AF, a significant association was found between the onset of POAF, CPB time, and plasmatic ORM levels measured in the operating theatre before surgical incision. Specifically, the addition of ORM to the predictive model significantly improved the ability to predict POAF during hospitalization after heart surgery. These results could be of help for a better understanding of POAF pathophysiology, adding valuable information to the inflammatory hypothesis, which is particularly associated with cardiac surgery. This information could be of use for the development of future targeted or personalized therapies. Patients with higher inflammatory status at baseline could have a lower threshold for the development of POAF during the post-operative period after cardiac surgery, making them more vulnerable to this condition.

### 4.1. Clinical and Biomarker Predictors of POAF

Among all the variables studied, advanced age has been one of the most consistent predictors of POAF according to previous data. Aging entails the accumulation of oxidative stress and reactive species leading to a progressive decline in the efficiency of tissues and organs [16]. In the human heart, the atria suffer from changes in their substrate such as alterations affecting the connective tissue, progressive dilatation, and electrical remodeling of atrial myocytes [17].

Several metabolic and humoral pathways are believed to act as intervening factors in the occurrence of AF. Such is the case of the autonomic nervous system, the renin-angiotensin-aldosterone system, and other less acknowledged routes, namely fibrosis or heart strain, and stretching. Nevertheless, pharmacological intervention with beta-blockers, ACE inhibitors, and mineralocorticoid receptor antagonists have been extensively studied for POAF prevention with mixed results [18].

Carbohydrate metabolism has been another intensive area of research for its relationship with AF. Lee et al. found a significant association between insulin resistance and AF risk, regardless of diabetes mellitus [19]. Moreover, in a population of patients with drug-refractory AF episodes who underwent pulmonary vein isolation, it was found to be a predictor of AF recurrence-inducing delays in left atrial conduction [20]. In our study, we could not find an association between insulin levels and POAF development, although higher insulin levels were more frequently found in the POAF group.

Epicardial fat is contemplated as an endocrine organ. Its secretome and differential expression of inflammatory and modulatory proteins was described as a possible substrate of POAF in patients undergoing coronary artery bypass surgery [21]. It has been shown that cholinergic activity can regulate the inflammatory secretome of this tissue [22]. This might explain the POAF reduction rate after botulin toxin injection on the epicardial fat pad from patients with paroxysmal AF found by Romanov et al. [23]. Local inflammatory cell infiltration and higher neutrophil or monocyte migration activity might be part of this mechanism [24]. In our study population, higher levels of adiposity marker (FABP4) were found in epicardial fat from patients who developed POAF. In the same line, our group has demonstrated higher adiposity in the epicardial stroma of patients with long-standing persistent AF [25] and the association of plasmatic FABP4 levels with atrial adiposity and recurrence of AF after catheter ablation.

A strong component of inflammation after cardiac surgery might be likely. Supporting this hypothesis, posterior left pericardiotomy has recently been shown to be effective for the reduction of POAF after coronary, valvular, or aortic surgery [26]. Surgical trauma, blood loss, and transfusion can contribute to the systemic inflammatory response. The beneficial effect of this technique is believed to be related to less mechanical compression and inflammation around the atria as the pericardiotomy allows the evacuation of fluid or thrombi to the left pleural space.

We studied alpha1-acid glycoprotein, also known as ORM. This is an acute-phase protein synthesized in the liver. It is related to a significant number of physiological functions, including binding and transporting molecules in the blood, modulating the immune system, regulating inflammation, and maintaining tissue homeostasis. Its concentration increases in response to tissue injury, infection, or systemic inflammation. This protein has been studied in other cardiac conditions, specifically in acute and chronic heart failure. Higher concentrations of ORM have been linked to worse outcomes in this population, even independently of NT-proBNP levels [27].

Moreover, the CPB is often employed to provide circulatory and respiratory support to the patient’s organs while the surgeons can perform their operation on a bloodless field. CPB induces an ischemia-reperfusion type injury and a complex inflammatory response. This may influence the post-operative course in the form of systemic and local tissue irritation [28], which may be dependent on how long the CPB and cross-clamp times are.

Patients undergoing open heart surgery face the consequences of not only their structural heart disease but also of surgical trauma and peri-operative care. Depending on their baseline inflammatory status, some patients may trigger POAF with insults of lesser intensity than others.

### 4.2. Clinical Relevance

Our findings complement other studies in the field of biomarkers and cardiac surgery. Although it is still premature for its implementation in routine clinical practice, there are promising data on the usefulness of serum biomarkers as tools for enhanced and personalized POAF risk stratification. We describe for the first time the predictive value of ORM as a risk factor for the development of POAF. This might have important implications for a better understanding of the physiopathology surrounding the inflammatory hypothesis, which is believed to have an important role in the field of cardiac surgery. ORM was not a predictor of POAF in this subset of male patients. However, those who developed POAF and those who did not have similar levels of ORM were physiological. We cannot rule out its additive predictive value in those patients with higher, and pathological levels, as we demonstrated in women. A larger study would help us to clarify this point.

Moreover, the results of the present study could also be of help for a tailored follow-up of patients at higher risk. Potentially, this subgroup of patients may benefit from preventive strategies such as pharmacological interventions that blunt the sympathetic response after cardiac surgery (preoperative prescription of anti-arrhythmic drugs or beta-blockers) or bi-atrial temporary pacing, which is believed to suppress automatic foci and reduce the dispersion of atrial refractory periods [29,30].

## 5. Limitations

The observational nature of the study may be subject to inherent bias. We included a small number of patients. Nevertheless, it is comparable to other studies performed in this setting, especially including some biomarkers. Although it was performed in a single center, several surgeons and anesthetists participated in the surgical process, so it is possible that some differences in the surgical technique or anesthetist regimes could have existed. Importantly, as patients are not under continuous ECG monitoring at the time of discharge to the surgical ward, exists the possibility that asymptomatic or subclinical episodes of AF (especially paroxysmal) might have been missed and therefore not included in our analyses. Nevertheless, all patients undergo daily ECG plus vital signs assessment up to at least three times per day.

Our study included a mixed population of patients with coronary and valvular heart disease. While valvular patients with extensive pre-operative structural changes were a minority (those requiring mitral intervention), we acknowledge that the specific characteristics of our patient population may limit the generalizability of our findings.

Although we studied other biomarkers and some of them were statistically significant in the univariate regression analysis, we finally did not include them in the multivariate model. This would have reduced significantly the number of subjects used to generate the regression model and therefore compromised the statistical significance of the rest of the predictor variables. Adipocyte and leukocyte biomarkers are less accessible and more costly than plasmatic biomarkers and they were not available for all patients, nevertheless, we consider that they could be of interest for further mechanistic studies.

## 6. Conclusions

In cardiac surgical patients, higher ORM at the moment of the surgical procedure was an independent risk factor for the development of POAF and its addition to EuroSCORE II and CPB time showed a significant improvement in risk stratification.

## Figures and Tables

**Table 1:** Baseline characteristics of the overall cohort.

Characteristics	Cohort (n = 123)
Age [years] mean ± SD	65.5 ± 10.3
Male (%)	93 (75.6%)
Diabetes mellitus (%)	39 (31.7%)
EuroSCORE II [%] mean ± SD	2.3 (4.9)
Anti platelet (%)	60 (48.8%)
Beta-blockers (%)	55 (44.7%)
ACEI-ARB (%)	66 (53.7%)
Mineralocorticoid receptor antagonist (%)	14 (11.4%)
Statin (%)	87 (70.7%)
Ischemic heart disease (%)	40 (32.5%)
Previous heart failure admission (%)	18 (14.6%)
Hemoglobin [g/dL] mean ± SD	13.7 ± 1.8
Creatinine [mg/dL] mean ± SD	1.17 ± 1.3
NTproBNP [pg/mL] mean ± SD	2022.1 ± 4096.2
LVEF [%] mean ± SD	56.1 ± 13.0
Left atrial size [cm^2^] mean ± SD	22.2 ± 5.7
TAPSE [mm] mean ± SD	21.7 ± 3.9
CPB time [min] mean ± SD	86.2 ± 81.3
Type of surgery (%)	Coronary bypass: 56 (45.5%)
	Aortic valve replacement: 46 (37.4%)
	Mitral valve replacement: 6 (4.9%)
	Other: 15 (12.2%)

**Table 2:** Post-operative course.

Complications during Peri-Operative Course	Cohort (n = 123)
Post operative atrial fibrillation	43 (34.9%)
Cardiac tamponade	2 (1.6%)
Bleeding requiring surgical re-exploration	2 (1.6%)
Renal replacement therapy	3 (2.4%)
Reintubation	3 (2.4%)
Death	2 (1.6%)

**Table 3:** Differences between patients who developed POAF or not.

	No POAF (n = 80)	POAF (n = 43)	*p*-Value
Age [years] mean ± SD	63.2 ± 11.0	68.77 ± 8.2	0.007
Male %	79.7	69.2	0.213
Diabetes mellitus %	36.5	17.9	0.041
Ischemic heart disease %	36.5	28.2	0.376
Previous heart failure admission %	12.2	23.1	0.132
CHADS2-VASc mean ± SD	3.17 ± 1.4	3.14 ± 1.3	0.915
EuroSCORE II [%] median (interquartile range)	1.1 (0.8–1.9)	1.77 (1.19–2.39)	0.004
LVEF [%] median (interquartile range)	60 (45–66)	60 (47–66)	0.631
Left atrial size [cm^2^] median (interquartile range)	21 (16–24)	24 (19–27)	0.014
TAPSE [mm] mean ± SD	21.7 ± 4.2	22.1 ± 3.4	0.631
Anti platelet %	35.4	14.2	0.188
Beta-blockers %	33.6	13.3	0.134
ACEI-ARB %	52.7	59.0	0.524
Mineralocorticoid receptor antagonist %	10.8	12.8	0.750
Statin %	77.0	59.0	0.053
Hematocrit [%] mean ± SD	41.2 ± 5.3	40.6 ± 5.1	0.601
Creatinine [mg/dL] mean ± SD	1.29 ± 1.7	0.96 ± 0.27	0.241
NTproBNP [pg/mL] median (interquartile range)	580 (178–2423)	766 (245–1684)	0.277
CPB time [min] median (interquartile range)	66 (0–118)	121 (72–166)	0.001
Adiposity markers on subcutaneous adipose tissue [a.u.]			
FABP4_SAT mean ± SD	2.24 ± 0.095	2.25 ± 0.089	0.626
CD36_SAT mean ± SD	2.02 ± 0.065	2.02 ± 0.041	0.561
Inflammatory cell markers on subcutaneous adipose tissue [a.u.]			
CD11b_SAT mean ± SD	1.67 ± 0.472	1.69 ± 0.0.58	0.261
CD16_SAT mean ± SD	1.69 ± 0.966	1.69 ± 0.096	0.649
DEFA3_SAT mean ± SD	1.80 ± 0.153	1.75 ± 0.134	0.321
CD14_SAT mean ± SD	1.79 ± 0.663	1.81 ± 0.098	0.827
CD3_SAT mean ± SD	1.69 ± 0.095	1.89 ± 0.043	0.960
CD68_SAT mean ± SD	1.75 ± 0.060	1.73 ± 0.023	0.159
Fibroblast cell markers on subcutaneous adipose tissue [a.u.]			
PREF1_SAT mean ± SD	1.61 ± 0.084	1.62 ± 0.096	0.970
COL1A2_SAT mean ± SD	1.92 ± 0.058	1.89 ± 0.043	0.224
Adiposity markers on epicardial adipose tissue [a.u.]			
FABP4_EAT mean ± SD	2.15 ± 0.140	2.17 ± 0.108	0.045
CD36_EAT mean ± SD	1.96 ± 0.076	1.96 ± 0.040	0.535
Inflammatory cell markers on epicardial adipose tissue [a.u.]			
CD11b_EAT mean ± SD	1.67 ± 0.416	1.67 ± 0.030	0.624
CD16_EAT median (interquartile range)	1.69 (1.62–1.81)	1.66 (1.64–1.73)	0.375
DEFA3_EAT mean ± SD	1.82 ± 0.136	1.77 ± 0.148	0.292
CD14_EAT mean ± SD	1.80 ± 0.062	1.80 ± 0.056	0.621
CD3_EAT mean ± SD	1.66 ± 0.076	1.67 ± 0.062	0.782
CD68_EAT mean ± SD	1.72 ± 0.072	1.71 ± 0.047	0.378
Fibroblast cell markers on epicardial adipose tissue [a.u.]			
PREF1_EAT mean ± SD	1.63 ± 0.072	1.63 ± 0.078	0.802
COL1A2_EAT mean ± SD	1.92 ± 0.050	1.90 ± 0.029	0.132
Plasma adiposity or metabolic markers [ng/mL]			
FABP4_plasma median (interquartile range)	27 (16–51)	34 (22–55)	0.205
Leptin_plasma median (interquartile range)	6 (3–13)	7.5 (3.3–16)	0.445
Insulin_plasma median (interquartile range)	0.3 (0.1–0.5)	0.33 (0.2–0.8)	0.098
Plasma inflammatory markers			
ORM_plasma [mg/mL] median (interquartile range)	1.04 (0.8–1.5)	1.48 (0.9–1.99)	0.015
c5a_plasma [ng/mL] median (interquartile range)	3.4 (2.0–4.9)	3.3 (2.1–5.1)	0.949
Plasma fibrosis markers [ng/mL]			
GDF15_plasma median (interquartile range)	1.2 (0.8–1.8)	1.4 (0.9–2.1)	0.294
Thrombospondin-2_plasma median (interquartile range)	13.2 (8–20)	15 (9.1–23.3)	0.375
Plasma atrial stretching [ng/mL]			
ANP_plasma median (interquartile range)	1.04 (0.8–1.5)	9.5 (5.5–14)	0.242
Circulating Neutrophils migratory activity and phenotype [a.u.]			
%CONTROL median (interquartile range)	27 (22–43)	24 (19–40)	0.270
%C5a median (interquartile range)	26 (17–39)	29 (12–49)	0.957
MPO_N median (interquartile range)	1.62 (1.59–1.65)	1.61 (1.57–1.71)	0.801
CD16_N median (interquartile range)	1.81 (1.72–1.85)	1.86 (1.79–1.91)	0.006
OLFM4_N median (interquartile range)	1.62 (1.58–1.66)	1.61 (1.53–1.82)	1.000
CXCR2_N median (interquartile range)	1.85 (1.80–1.89)	1.88 (1.83–1.92)	0.057
NGAL_N median (interquartile range)	1.65 (1.58–1.71)	1.69 (1.61–1.82)	0.063
ICAM_N median (interquartile range)	1.71 (1.67–1.75)	1.68 (1.66–1.77)	0.593
MMP9_N median (interquartile range)	1.79 (1.75–1.83)	1.79 (1.76–1.87)	0.315
S100A9_N median (interquartile range)	2.16 (2.07–2.24)	2.16 (2.05–2.23)	0.947
SELL_N median (interquartile range)	1.92 (1.89–1.95)	1.94 (1.90–1.97)	0.297
CXCR4_N median (interquartile range)	1.91 (1.86–1.97)	1.89 (1.83–1.94)	0.218
LF_N median (interquartile range)	1.68 (1.62–1.72)	1.65 (1.61–1.73)	0.751
DFA3_N median (interquartile range)	1.87 (1.80–1.96)	1.83 (1.79–1.91)	0.293
CD11b_N median (interquartile range)	1.71 (1.70–1.76)	1.72 (1.69–1.77)	0.450
CD88_N median (interquartile range)	1.77 (1.70–1.85)	1.76 (1.71–1.83)	0.867
Circulating monocytes phenotype [a.u.]			
MPO_M median (interquartile range)	1.61 (1.58–1.70)	1.61 (1.57–1.64)	0.505
CD16_M median (interquartile range)	1.81 (1.73–1.89)	1.76 (1.71–1.85)	0.320
OLFM4_M median (interquartile range)	1.65 (1.58–1.75)	1.58 (1.57–1.66)	0.115
CXCR2_M median (interquartile range)	1.81 (1.71–1.87)	1.78 (1.71–1.87)	0.682
NGAL_M median (interquartile range)	1.68 (1.60–1.77)	1.63 (1.61–1.65)	0.179
ICAM_M median (interquartile range)	1.65 (1.62–1.70)	1.67 (1.62–1.71)	0.740
MMP9_M median (interquartile range)	1.76 (1.71–1.82)	1.74 (1.69–1.81)	0.360
S100A9_M median (interquartile range)	2.1 (2.033–2.19)	2.17 (2.08–2.21)	0.090
SELL_M median (interquartile range)	1.89 (1.86–1.94)	1.89 (1.85–1.96)	0.711
CXCR4_M median (interquartile range)	1.86 (1.79–1.89)	1.86 (1.80–1.94)	0.711
LF_M median (interquartile range)	1.71 (1.66–1.74)	1.69 (1.64–1.71)	0.129
DFA3_M median (interquartile range)	1.88 (1.81–1.94)	1.86 (1.82–1.89)	0.407
CD11b_M median (interquartile range)	1.70 (1.68–1.74)	1.71 (1.68–1.72)	0.711
CD88_M median (interquartile range)	1.76 (1.70–1.85)	1.72 (1.67–1.82)	0.333

**Table 4:** Sex differences between patients who developed POAF or not.

	Female Sex (n = 30)			Male Sex (n= 93)		
	No POAF (n = 16)	POAF (n = 14)	*p*-Value	No POAF (n = 64)	POAF (n = 29)	*p*-Value
Age [years] mean ± SD	66 ± 13	72 ± 6	0.276	64 ± 10	65± 10	0.252
Diabetes mellitus (%)	5 (33)	0 (0)	0.082	22 (37)	7 (26)	0.273
Ischemic heart disease (%)	5 (33)	1 (8)	0.179	22 (37)	10 (37)	0.901
Previous heart failure admission (%)	0 (0)	4 (33)	0.031	9 (15)	5 (19)	0.473
CHADS2-VASc mean ± SD	3.5 ± 1.6	3.2 ± 1.1	0.666	4.1 ± 1.4	3.1 ± 1.4	0.354
EuroSCORE II [%] median (interquartile range)	0.9 (0.8–1.4)	1.8 (1.2–2.7)	0.064	1.2 (0.8–1.8)	1.7 (1.2–2.2)	0.025
LVEF [%] median (interquartile range)	60 (47–67)	65 (62–71)	0.120	59 (45–65)	57 (43–65)	0.533
Left atrial size [cm^2^] median (interquartile range)	19 (16–24)	24 (22–25)	0.083	21 (16–24)	23 (18–31)	0.065
TAPSE [mm] mean ± SD	21 ± 3.4	21 ± 3.7	0.928	22 ± 4	23 ± 3	0.187
Anti platelet (%)	6 (67)	3 (25)	0.343	45 (76)	18 (67)	0.709
Beta-blockers (%)	7 (47)	3 (25)	0.079	31 (52)	12 (44)	0.124
ACEI-ARB (%)	6 (40)	7 (58)	0.177	33 (56)	16 (59)	0.959
Mineralocorticoid receptor antagonist (%)	1 (7)	1 (8)	0.361	7 (12)	4 (15)	0.557
Statin (%)	12 (80)	5 (42)	0.079	45 (76)	18 (67)	0.492
Hematocrit (%) mean ± SD	38 ± 3.8	41 ± 3.7	0.063	42 ± 5.3	41.60 ± 5.6	0.223
Creatinine [mg/dL] ± SD	1.2 ± 1.9	0.8 ± 0.1	0.783	1.3 ± 1.7	1.2 ± 1.4	0.628
NTproBNP [pg/mL] median (interquartile range)	2923 (2503–3474)	745 (477–2122)	0.010	401 (145–1262)	829 (197–1600)	0.285
CPB time [min] median (interquartile range)	68 (0–93)	143 (96–163)	0.007	63 (0–133)	103 (0–168)	0.053
Adiposity markers on epicardial adipose tissue [a.u.]						
FABP4_EAT mean ± SD	2.22 ± 0.09	2.23 ± 0.04	0.770	2.13 ± 0.14	2.13 ± 0.11	0.238
CD36_EAT mean ± SD	1.97 ± 0.03	1.98 ± 0.02	0.464	1.97 ± 0.08	1.94 ± 0.04	0.855
Inflammatory cell markers on epicardial adipose tissue [a.u.]						
CD11b_EAT mean ± SD	1.69 ± 0.03	1.67 ± 0.03	0.464	1.67 ± 0.04	1.67 ± 0.03	0.765
CD16_EAT median (interquartile range)	1.8 (1.7–1.8)	1.6 (1.6–1.7)	0.008	1.7 (1.6–1.8)	1.7 (1.6–1.8)	0.551
DEFA3_EAT mean ± SD	1.94 ± 0.07	1.74 ± 0.13	0.013	1.80 ± 0.13	1.79 ± 0.16	0.976
CD3_EAT mean ± SD	1.70 ± 0.03	1.68 ± 0.08	0.380	1.65 ± 0.08	1.66 ± 0.04	0.626
CD14_EAT mean ± SD	1.86 ± 0.03	1.78 ± 0.05	0.019	1.79 ± 0.06	1.81 ± 0.05	0.417
CD68_EAT mean ± SD	1.69 ± 0.05	1.71 ± 0.04	0.464	1.73 ± 0.07	1.70 ± 0.05	0.175
Fibroblast cell markers on epicardial adipose tissue [a.u.]						
PREF1_EAT mean ± SD	1.67 ± 0.06	1.67 ± 0.08	0.884	1.61 ± 0.07	1.60 ± 0.06	0.683
COL1A2_EAT mean ± SD	1.96 ± 0.04	1.89 ± 0.03	0.013	1.91 ± 0.05	1.90 ± 0.03	0.791
Plasma adiposity or metabolic markers [ng/mL]						
FABP4_plasma median (interquartile range)	50 (11–69)	47 (33–75)	0.626	26 (16–47)	31 (16–50)	0.645
Leptin_plasma median (interquartile range)	6 (4–18)	14 (7–26)	0.071	6 (3–12)	4 (2–9)	0.534
Insulin_plasma mean ± SD	0.51 ± 0.87	0.45 (0.17–0.89)	0.207	0.31 (0.1–0.5)	0.32 (0.18–0.82)	0.233
Plasma inflammatory markers						
ORM_plasma [mg/mL] median (interquartile range)	1.0 (0.7–1.2)	1.9 (1.4–2.1)	0.001	1.0 (0.8–1.5)	1.25 (0.8–1.7)	0.445
c5a_plasma [ng/mL] median (interquartile range)	3 (1.6–4.5)	4 (2.5–4.9)	0.435	3.4 (2.2–5.1)	3.0 (1.6–1.7)	0.856
Plasma fibrosis markers (ng/mL)						
GDF15_plasma median (interquartile range)	1.2 (0.8–1.6)	1.3 (1.0–2.1)	0.440	1.2 (0.7–1.8)	1.5 (0.9–2.1)	0.486
Thrombospondin-2_plasma median (interquartile range)	18 (9–22)	15 (7–22)	0.845	13 (7–19)	14 (9–25)	0.304
Plasma atrial stretching (ng/mL)						
ANP_plasma median (interquartile range)	7 (5–10)	11 (615)	0.123	7 (7–13)	8 (5–14)	0.661

**Table 5:** Logistic regression analyses. (a) Univariate logistic regression analysis. (b) Multivariate logistic regression analysis. (c) Multivariate logistic regression analyses (Women only).

**(a)**			
**Variable**	**Odds Ratio**	**95% Confidence Interval (Lower-Upper)**	***p* Value**
EuroScoreII (%)	1.007	(0.932–1.087)	0.869
CPB TIME (min)	1.007	(1.002–1.013)	0.006
PLASMA ORM (mg/mL)	2.207	(1.102–4.422)	0.026
**(b)**			
**Variable**	**Odds Ratio**	**95% Confidence Interval (Lower-Upper)**	***p* Value**
EuroScoreII (%)	0.953	(0.872–1.041)	0.289
CPB TIME (min)	1.008	(1.002–1.013)	0.005
PLASMA ORM (mg/mL)	2.636	(1.206–5.761)	0.015
**(c)**			
**Variable**	**Odds Ratio**	**95% Confidence interval (lower-upper)**	***p* Value**
EuroScoreII (%)	0.749	(0.303–1.851)	0.531
CPB TIME (min)	1.023	(1.000–1.047)	0.05
PLASMA ORM (mg/mL)	2.639	(1.455–4.788)	0.027

## Data Availability

The data that support the findings of this study are available from the corresponding author, upon reasonable request.
